# A comparative analysis of metal transportomes from metabolically versatile *Pseudomonas*

**DOI:** 10.1186/1756-0500-1-88

**Published:** 2008-09-24

**Authors:** Adhikarla Haritha, Agnes Rodrigue, Pamarthi Maruthi Mohan

**Affiliations:** 1 Department of Biochemistry, Osmania University, Hyderabad, India; 2 Université de Lyon, Université Lyon 1, INSA de Lyon, CNRS, UMR5240, Microbiologie Adaptation et Pathogénie, Villeurbanne, F-69621, France

## Abstract

**Background:**

The availability of complete genome sequences of versatile *Pseudomonas *occupying remarkably diverse ecological niches enabled to gain insights into their adaptative assets. The objective of this study was to analyze the complete genetic repertoires of metal transporters (metal transportomes) from four representative *Pseudomonas *species and to identify metal transporters with "Genomic Island" associated features.

**Methods:**

A comparative metal transporter inventory was built for the following four *Pseudomonas *species: *P*.*putida (Ppu) *KT2440, *P.aeruginosa *(*Pae*) PA01, *P.fluorescens *(*Pfl*) Pf-5 and *P.syringae *(*Psy*)*pv.tomato DC3000 *using TIGR-CMR and Transport DB. Genomic analysis of essential and toxic metal ion transporters was accomplished from the above inventory. Metal transporters with "Genomic Island" associated features were identified using Islandpath analysis.

**Results:**

Dataset cataloguing has been executed for 262 metal transporters from the four spp. Additional metal ion transporters belonging to NiCoT, Ca P-type ATPase, Cu P-type ATPases, ZIP and MgtC families were identified. In *Psy *DC3000, 48% of metal transporters showed strong GI features while it was 45% in *Ppu *KT2440. In *Pfl *Pf-5 and *Pae *PA01 only 26% of their metal transporters exhibited GI features.

**Conclusion:**

Our comparative inventory of 262 metal transporters from four versatile *Pseudomonas *spp is the complete suite of metal transportomes analysed till date in a prokaryotic genus. This study identified differences in the basic composition of metal transportomes from *Pseudomonas *occupying diverse ecological niches and also elucidated their novel features. Based on this inventory we analysed the role of horizontal gene transfer in expansion and variability of metal transporter families.

## Findings

### Background

Metal ions play a variety of critical roles in the prokaryotic metabolism. Biological management of metal ions is accomplished by a complex interplay between metal ion transporters (transmembrane importers, transmembrane exporters) and their regulatory components [1]. In the post-genomic era, genetic landscapes of the organisms have unveiled the hidden facets of metal ion transporters and associated regulatory cascade thereby paving way for study of their metal transportomes.

The genus *Pseudomonas *is one of the most versatile and ecologically significant groups of bacteria on the planet that thrives well in metal rich biotopes. In recent studies, the unexpected capacity of *Ppu *KT2440 and *Ppu *CD2 genomes to endure exposure to heavy metals has been unraveled [2,3]. In *Ppu *KT2440 metal transporters acquired through HGT were also reported [4]. However, a systematic study of complete metal transportomes is unavailable for any of these *Pseudomonas *species. The availability of complete genome sequences for saprophytic *P.putida *(*Ppu*) KT2440, commensalistic *P.fluorescens *(*Pfl*) Pf-5, pathogenic *P.aeruginosa *(*Pae*) PA01 and *P.syringae *pv. *tomato *(*Psy) DC*3000 [5-8] provided an opportunity for analyzing their complete repertoire of metal transporters along with the role of HGT in their acquisition.

## Methods

### Compilation of metal transporter inventory

The genome sequence information of the four sequenced *Pseudomonas *spp *Ppu *KT2440, *Pae *PA01, *Pfl *Pf-5 and *Psy *DC3000 employed in this study were selected from TIGR-CMR database [9]. TransportDB, a relational database describing the predicted cytoplasmic membrane transport protein complement for organisms was used as a substrate for building the metal transportomes [10]. In order to search for homologs of a transporter family the best-annotated hit was used in a subsequent BLASTP search against the four genomes and retrieved the members. PFAM domain search was also carried out with the retrieved homologs, using CLC HMM algorithm [11] and PFAM database [12]. Transmembrane helix prediction for the membrane transporters was performed using TMHMM version 2.0 [13]. Genomic location and gene organization of transporters were identified from TIGR-CMR database.

### Protein sequence analysis

CLC protein workbench has been employed to analyse the metal transporter proteins [11]. Genchek™ Graphical Viewer was used to represent GTPase signature motifs G1-G5 in the alignment generated for FeoB_*Eco *_and FeoB_*Pae*_.

### Phylogenetic analysis

Phylogenetic analysis was performed with the characterised prototypes using CLC protein workbench [11]. For this analysis, multiple sequence alignments (MSA) were produced using progressive alignment algorithm. The generated pair wise alignments were used for finding the evolutionary distance between the pairs. Pair wise distances thus calculated was used to create a phylogenetic tree-employing neighbour joining (NJ) algorithm with 1000 bootstrap replicates.

### Sequence Clusters

Metal transporters located in sequence clusters, were identified by cluster viewer from PEDANT database [14]. Cluster viewer aided us in identifying clusters of genes in the same organism that are paralogs. Sequence clusters thus obtained provided us with data regarding the metal transporters that have a BLAST similarity score greater than 45 bits in PSI-BLAST and also possess a similar Pfam domain with a significant E-value (typically 0.001). This tool helped us in determining paralogous genes of a family and aided in phylogenetic analyses.

### Island Path Analysis

Island Path Analysis was used for the detection of metal transporters acquired through HGT [15]. After generating the complete inventory of metal transporters, we inspected the genomes of four *Pseudomonas *species with island path software (IPA version 1.0 tool) for the identification of those transporters located in GI's or exhibiting GI associated features like anomalous %G+C, dinucleotide bias above 1 STD DEV, presence of RNA genes (tRNA, rRNA genes) and mobility genes (transposons, insertion sequences). A putative GI can be identified with certainty by the presence of eight or more consecutive ORF's with dinucleotide bias alone or dinucleotide bias plus a mobility gene in proximity.

## Results and discussion

### From genome to metal transportome in Pseudomonas

Based on the global features of the four *Pseudomonas *genomes we could draw a comparison among the genome size, number of genes, transporter proteins and total number of metal transporters (Table 1). Among the four spp, *Pfl *has the largest metal transporter inventory (75 metal ion transporters) while *Psy *has the smallest (54 metal ion transporters). Other pathogenic species, *Pae *has more number of genes and transporter proteins but relatively less metal transporters (63 metal ion transporters) than *Ppu *(70 metal ion transporters).

**Table 1:** Global features of four representative *Pseudomonas *genomes

**Topology**	***P.putida KT2440***	***P.aeruginosa PA01***	***P.fluorescens Pf5***	***P.syringae DC3000***
Genome size (bp)	6,181,862	6,264,403	7,074,893	6,538,260
G+C content (%)	61.6	66.5	63.3	58.3
Total no: of genes	5516	5565	6230	5843
Total Transporter Proteins	386	423	475	322
Total Metal Transporters	70	63	75	54

Based on the TransportDB [10] we compiled the metal transportomes for the entire complement of alkali/alkaline earth metals (Na^+^, K^+^, Ca^2+^, Mg^2+^), transition metals (Zn^2+^, Mn^2+ ^Cu^+^, Ag^+^, Mo^2+^, Fe^2+^, Fe^3+^, Ni^2+^, Co^2+^) and heavy metals (Cr^3+^, As^3+^, Pb^2+^, Cd^2+^) in *Pseudomonas *(Additional files 1, 2, 3, 4). These transportomes furnish information for class/family/subfamily, transporter classification number (T.C.No), TIGR locus, protein name, genomic location, orientation, length of the protein, TMD's, substrate and the predicted role of the above metal transporters. Sequence cluster analysis helped in identifying paralogs for the transporter families (see Additional file 5). In this study we employed the Transporter Classification system-TC system for systematic classification of *Pseudomonas *metal transporters into the following four groups: ATP-dependent (ABC superfamily, P-type ATPase super family) Ion-channels, Secondary transporters and Unclassified. A comparative analysis depicting the basic differences in the composition of transporter classes and metal transporter families from the four *Pseudomonas *species is represented in Table 2 (This table also provides expanded names of the transporter families).

**Table 2:** Comparative Analysis of Metal transporters from *P.aeruginosa *PA01, *P.fluorescens *Pf-5, *P.putida *KT2440 and *P.syringae *DC3000

**Metal Transporter Type/Family**	***Number of Transporters***			
	
	***P.aeruginosa PAO1***	***P.fluorescens Pf5***	***P.putida KT2440***	***P.syringae Pv tomato DC 3000***
**I ATP Dependent**	17 (26.98%)	18 (24. 32%)	17 (24.28%)	16 (29.62%)
The ATP-binding cassette (ABC) super family	10	10	10	11
The P-type ATPase (P-ATPase) super family	7	8	7	5
**II. Ion Channels**	6 (9.52%)	5 (6.66%)	4 (5.71%)	7(12.96%)
The CorA metal ion Transporter family (MIT family)	3	3	3	4
Small-conductance mechanosensitive ion channel (MSCS) family	1	1	-	1
The voltage-gated ion channel superfamily (VIC)	2	1	1	2
**III Secondary Transporters**	36 (57.14%)	47 (62.66%)	45 (64.28%)	28 (51.85%)
The Alanine/Glycine: Cation Symporter (AGCS) family	3	1	1	1
The Arsenite-Antimonite (ArsB) efflux family	1	2	2	1
The Bile acid: Na^+ ^symporter (BASS) family	3	2	2	1
The Ca^2+^: Cation Antiporter (CaCA) family	-	2	1	2
The cation diffusion facilitator (CDF) family	3	4	2	1
The Chromate Ion Transporter (CHR) family	1	1	1	-
The Citrate Mg^2+^: H^+ ^(CitM) Citrate-Ca^2+^: H^+ ^(CitH) Symporter (CitMHS) family	-	3	2	1
The Monovalent cation: Proton Antiporter-1 (CPAI) family	5	5	4	3
The Monovalent cation: Proton Antiporter-2 (CPA2) family	3	4	4	3
The Monovalent cation: (K^+^or Na^+^) Proton Antiporter-3 (CPA3) family	1	1	1	1
The Dicarboxylate/AminoAcid: cation (Na^+^/H^+^) Symporter (DAACS) family	1	3	2	-
The Divalent Anion: Na+ symporter (DASS) family	1	1	1	-
The glutamate: Na^+ ^symporter (ESS) family	1	1	1	
The K^+ ^Uptake Permease (KUP) family	1	1	1	1
The Malonate: Na^+^symporter (MSS) family	1	1	-	-
The NhaA Na^+^:H^+ ^Antiporter (NhaA) family	-	1	2	2
The NhaB Na^+^: H^+ ^Antiporter (NhaB) family	1	1	1	-
The Ni^2+^-Co^2+^Transporter (NicoT) family	-	-	1	1
The Metal Ion (Mn^2+^- Fe^2+^) Transporter (Nramp) family	2	1	-	3
The Neurotransmitter sodium symporter (NSS) family	-	1	-	1
PNaS family: The Phosphate sodium symporter family	2	2	1	1
				
The Resistance-Nodulation Cell Division (RND) super family	2	2	6	-
The solute: sodium symporter (SSS) family	2	4	5	3
The K+ transporter (Trk) family	1	1	2	1
The Zinc (Zn^2+^)-Iron (Fe^2+^) Permease (ZIP) family	1	2	2	1
**IV Unclassified**	4 (6.35%)	5 (6.66%)	4 (5.71%)	3 (5.55%)
The Ferrous Iron uptake (FeoB) family	1	-	-	-
The Iron/Lead Transporter (ILT) super family	1	2	1	2
The Mg^2+^Transporter E (MgtE) family	1	1	1	1
The Mg^2+^Transporter C (MgtC) family	1	2	2	-

**Total**	**63**	**75**	**70**	**54**

### Salient Features of Pseudomonas metal transportomes

Apart from executing the dataset cataloguing, our analysis of *Pseudomonas *databases identified additional metal transporters belonging to Ca, ZIP, NiCoT, MgtC, ABC (copper), P-type ATPase (copper) families. Distinct features of transporters belonging to CPA3, CaCA, ZnuABC, MntABC, ZntA, Nramp, FeOB, OFeT, NikABC, Chr, PbrT, RND, CDF families and ABC super family were also reported.

Search for primary ATP-dependent calcium pumps in *Pseudomonas *using two characterized members (SP1551 and SP1623) of *S.pneumoniae *[16] identified a Ca P-type ATPase (PA1429) in *Pae *alone with no reciprocal BLAST hits from other three species.

ZIP family of transporters in *Pseudomonas *were retrieved using the experimentally characterized transporter ZupT_*Eco *_[17]. *Psy *and *Pae *have single entities PA4467 and PSPTO_2053 while paralog expansion is seen for *Ppu *(PP_1836, PP_0947) and *Pfl *(PFL_4718, PFL_0910).

Using the experimentally characterized efflux member RcnA_*Eco *_of NiCoT subfamily 2 [18] as a BLAST query against *Pseudomonas *databases, we identified two new efflux members PP_2968 (RcnA_*Ppu*_) and PSPTO_4280 (RcnA_*Psy*_).

The experimentally characterized MgtC family transporter *Pae *(PA4635) [19] was used in the BLAST P analysis to identify the following MgtC homologs: PP_3244, PP_3541 (*Ppu*), PFL_4077, PFL_2871 (*Pfl*), PA4635 (*Pae*). No hits were identified for this family in *Psy*.

*Pae *uniquely harbours an ABC copper transporter PA3393 that is related to periplasmic copper binding protein NosD in *Rhizobium meliloti *whose function was presumed to insert copper into the exported reductase apoenzyme (NosZ) [20]. All the CuPATP1 transporters of *Pseudomonas *(PFL_0710, PP_0586, PA3920, PSPTO_0750) were found to be the best BLAST hits for experimentally characterized CopA_*Ehi*_, while the other group of CuPATP2 transporters (PP_4261, PA1549, PFL_1915, PSPTO_1996) did not show significant BLAST identities with the characterized proteins of CopA, CopB.

CPA3 family members constituting multicomponent K^+ ^efflux system are present in all the three species except in *Pae *where this component is involved in sodium ion homeostasis. *Ppu *(PP_2225 to PP_2230) and *Pfl *Pf-5 (PFL_2606 to PFL_2611) have six component K^+ ^efflux systems while it is a five-component system in *Psy*.

**Figure 1:**
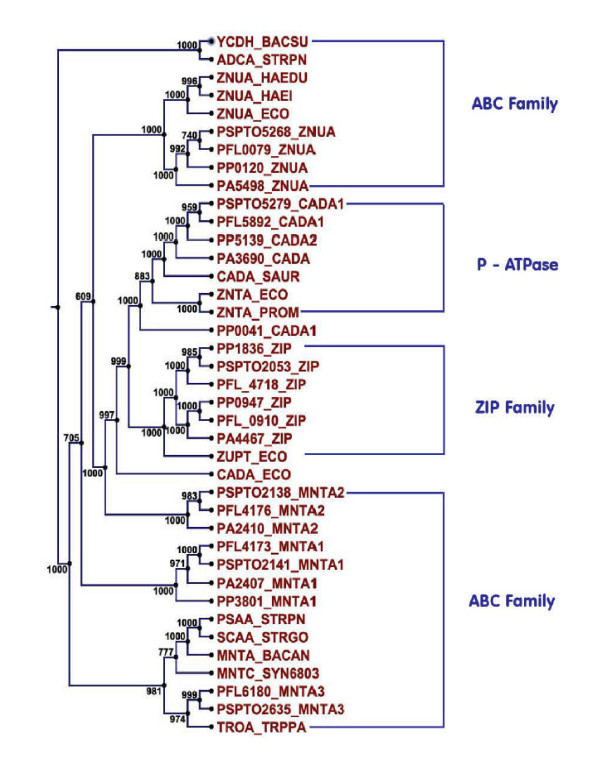
**Phylogenetic tree for Zinc/Manganese transporters using neighbour joining method with 1000 bootstrap replicates.** It depicts the grouping of various orthologs of *Pseudomonas *species from three families (ABC, P-type ATPase and ZIP families). BACSU, *Bacillus subtilis*; STRPN, *Streptococcus pneumoniae*; HAEDU, *Haemophilus ducreyi*; HAEIN, *Haemophilus influenzae*; ECO, *Escherichia coli*; PROM, *Proteus mirabilis; *STRPN, *Streptococcus pneumoniae; *STRGO, *Streptococcus gordonii; *BACAN, *Bacillus anthracis*; SYN6803, *Synechococystis *sp strain PCC6803; TRPPA, *Treponema pallidum*.

CaCA transporters i.e. secondary calcium transporters in *Pfl *and *Psy *(PFL_0722 and PSPTO_0764) are annotated as Ca^2+^/H^+ ^(ChaA) antiporters that exhibit significant homology to ChaA of *E.coli *[21]. Proteins resembling mammalian Na^2+^/Ca^2+^exchangers (YrbG) [22] are present in *Ppu *(PP_0928), *Pfl *Pf-5 (PFL_0891) and *Psy *(PSPTO_4477). *Pae *PA01 lacks a distinct homologue for CaCA transporter. Representative Zinc transporters of *Pseudomonas *species are presented in table 3. ABC zinc transporters (ZnuABC) that recognize Zn^2+ ^as their substrate and ABC manganese transporters (MntABC) that recognize Mn^2+^/Zn^2+^, were included in the phylogenetic profiling to study their overlapping specificities (Figure 1). Search for ZntA homologs in the *Pseudomonas *genomes using experimentally characterized ZntA_*Eco *_[23] resulted in identification of CadA as the closest homologue with 37% identity from all the four species. However in *Pseudomonas*, only CadA proteins with five (PFL_5892, PSPTO_5279, PA3690) or six TMD's (PP_0041, PP_5139, PA2435) were identical to ZntA. The other CadA proteins with seven TMD's (PFL_6191, PSPTO_5532) did not show any significant identity in BLAST analysis (less than 24% identity) and appear to be unrelated to ZntA.

**Table 3:** Representatives of Zinc Transporters from *P.putida *KT2440, *P.aeruginosa *PA01, *P.fluorescens *Pf-5 and *P.syringae *DC3000

**Transporter protein**	**Functional family**	**Transport direction**	***Pseudomonas *species**	**Number of Paralogs/Orthologs**
ZnuABC	ABC ATPase	Uptake	*Pputida*	2
			*P.aeruginosa*	3
			*P.fluorescens*	4
			*P.syringae*	4
ZupT	ZIP chemiosmotic (2^0 ^transporters)	Uptake	*P.putida*	2
			*P.aeruginosa*	1
			*P.fluorescens*	2
			*P.syringae*	1
CorA	MIT family (Ion channels)	Uptake	*P.putida*	1
CadA	P-type ATPases	Efflux	*P.putida*	2
			*P.aeruginosa*	1
			*P.fluorescens*	1
			*P.syringae*	1
CzcCBA	RND chemiosmotic (2^0 ^transporters)	Efflux	*P.putida*	2
			*P.aeruginosa*	1
			*P.fluorescens*	1

CzcD	CDF chemiosmotic (2^0 ^transporters)	Efflux	*P.putida*	1
			*P.fluorescens*	1

**Figure 2:**
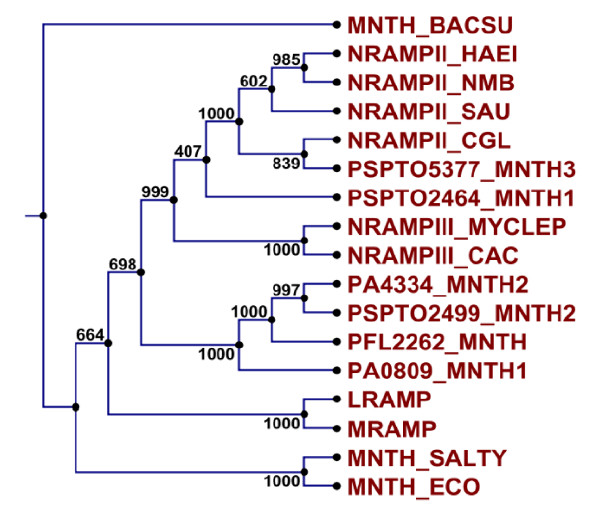
**Phylogenetic tree for Nramp family of manganese transporters using neighbour joining method with 1000 bootstrap replicates.** It depicts the grouping of various orthologs of *Pseudomonas *species within this family. BACSU, *Bacillus subtilis*; HAEI, *Haemophilus influenzae; *SAU, *Staphylococcus aureus*; CGL, *Corynebacterium glutamicum; *MYCLEP, Mycobacterium leprae; CAC, *Corynebacterium acetylicum*; SALTY, *Salmonella typhimurium*; ECO, *Escherichia coli*.

Only one Nramp member, MntH (PFL_2262) was observed for *Pfl *while *Pae *has two members MntH1 (PA0809), MntH2 (PA4334). *Psy *was found to possess three distinct members MntH1 (PSPTO_2464), MntH2 (PSPTO_2499) and MntH3 (PSPTO_5377) in this family. No conserved motifs characteristic for Nramp super family were observed for PSPTO_5377. However, phylogenetic analysis with Nramp family members confirmed its position in this family (Figure 2). Analysis of Lineage Specific Regions (greater than 2 Kb, enriched in mobile genetic elements and has genes specific to *Psy *DC3000) of *Psy *showed that PSPTO_5377 is the only metal transporter located in its LSR's (LSR no: 42).

**Figure 3:**
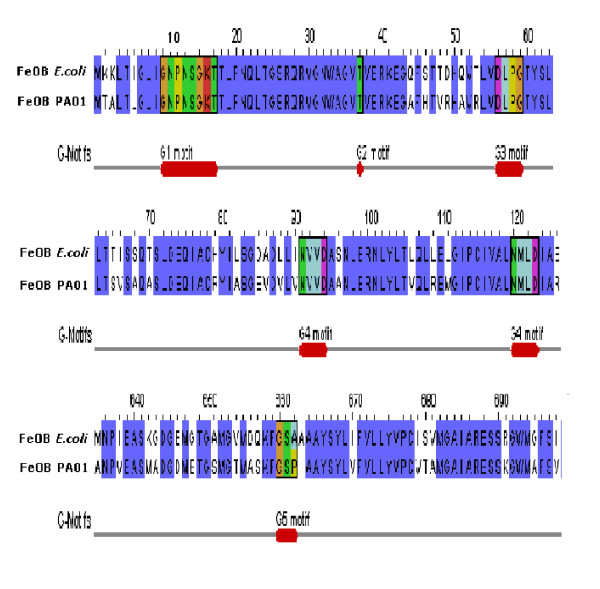
Multiple sequence alignment of FeoB transporters from *Escherichia coli *and *Pseudomonas aeruginosa *PA01 depicting conserved GTPase signature motifs (G1–G5).

Three types of iron transporters (ABC, FeOB and OFeT) were found in the *Pseudomonas *species with the restriction of FeOB transporter (PA4358) to *Pae *and OFeT transporter (PFL_3255) to *Pfl *(Figure 3). Based on the previous reports of GTPase motifs in FeoB_*Eco *_[24] highly conserved G1-G5 GTPase motifs were present in FeoB_*Pae *_(Figure 3). In OFeT_*Pfl *_two REXXE motifs (^10^REGIE^14^, ^129^REGLE^133^) were identified basing on the characterised prototype YcdN_*Eco *_[25]. No iron transporters were reported for pathogenic *Borrelia burgdorferi *and *Treponema pallidum *while we reported substantial number of iron transporters for the pathogenic *Pae *and *Psy *(six and four respectively).

In *Ppu *and *Psy *there are high affinity nickel uptake systems (Nik) of ABC family whose PBP's (PP_3342 and PSPTO_3088) belong to nickel/peptide/opine ABC transporter family. Two IMP's were identified for each of the two Nik systems, in Nik_*Ppu *_(PP_3343, PP_3344) and Nik_*Psy *_(PSPTO_3089, PSPTO_3090). They displayed a characteristic transmembrane topology of five (PSPTO_3089, PP_3344) and six TMD's (PSPTO_3090, PP_3343).

Chromate transporters of Chr family mediating chromate resistance are present in all the three *Pseudomonas *species (PP_2556, PA4289, PFL_3149) except *Psy*. PbrT members conferring lead resistance are also present in all the four *Pseudomonas *species (PA5248, PFL_5990, PP_0180). As an exception *Psy *possess two PbrT systems, PbrT1 and PbrT2 (PSPTO_0141, PSPTO_3596). PbrT2_*Psy *_did not show the conserved motifs found in other lead transporters of this group. Uniquely there is one ABC family member for Pb in *Ppu *(PP_5165) with no existing sequence homology to PbrT_*Ppu*_.

The best-characterized heavy metal resistant strains *R.metallidurans *have eight HME-RND proteins on chromosome. In comparison, *Ppu *has six RND chromosomal determinants (PP_0043, PP_2410, PP_3302, PP_5173, PP_1517, PP_5387), *Pfl *and *Pae *have two determinants each (PFL_2558, PFL_5218 PA0158, PA2520) while no RND determinant was identified in *Psy*. *Pfl *Pf-5 has a maximum of four CDF transporters (PFL_2508, PFL_5419, PFL_0604, PFL_5222) while they are three in *Pae *PA01 (PA0397, PA1297, PA3963) and two in *Ppu *KT2440 (PP_0026, PP_4774). However *Psy *DC3000 has only one distinct ortholog (PSPTO_4790) in this family.

Analysis of ABC super family members from the above inventory unravelled a distinct transmembrane topology for their integral membrane proteins (IMP's) that varied with the metal ion specificity of their associated PBP's. Accordingly, five/six TMD's were observed for nickel transporters, twelve for iron transporters, seven for zinc, eight for manganese and five for molybdenum

### Multiplicity of paralogs in metal transporter families

*Pseudomonas *species has multiple paralogs for most of the metal ion transporters that are briefed out in Additional table 5. Some of the significant features of paralog distribution are highlighted in this section.

Sodium ion transporters constitute the bulk of secondary transporters and has maximum number of paralogs distributed in different families (Table 4).

**Table 4:** Distribution of Sodium transporters in *P.putida *KT2440, *P.aeruginosa *PA01, *P.fluorescens *Pf-5 and *P.syringae *DC3000.

**Family**	***P.putida *KT2440**	***P.aeruginosa *PA01**	***P.fluorescens *Pf-5**	***P.syringae *DC3000**
AGCS	1	3	1	1
BASS	2	3	2	1
CaCA	1	-	1	1
CPA1	4	5	5	3
CPA2	1	1	2	1
CPA3	-	1	-	-
DAACS	2	1	3	-
DASS	1	1	1	-
ESS	1	1	1	-
MSS	-	2	2	-
NhaA	2	-	1	2
NhaB	1	1	1	-
NSS	-	-	1	1
PNAS	1	2	2	1
SSS	5	2	4	3

Total	22	23	27	14

A multitude of K^+ ^transporters are present in *Pseudomonas *species among which Kdp system (in *Pfl*), VIC super family (in *Pae *and *Psy*), CPA2 family (in *Ppu, Pae, Pfl*, *Psy*) and Trk family (in *Ppu*) possessed paralogs.

All the four *Pseudomonas *species were found to possess paralogs for PBP's of ABC Zn^2+ ^and Mn^2+ ^transporters. *Psy *alone has paralogs for PBP of ABC Mo^2+ ^transporter.

*Ppu *has two paralogs for CitMHS family, which are presumed to facilitate transport of Citrate-Mn^2+ ^complex while in *Pfl *this family has three paralogs with specificity for Mg^2+^/citrate.

All the four *Pseudomonas *species have paralogs for MIT family and P-type ATPases. Except for *Psy *all the three species possessed paralogs for RND and CDF families.

*Psy *and *Pfl *have paralogs for ILT family, mediating lead transport alone in the former while both ferrous iron and lead transport are mediated in the later.

In *Ppu *ArsB family has two paralogs that are part of separate arsRBCH operons while in *Pfl *out of the two paralogs (PFL_2185, PFL_3288) only former is located within an arsRBCH operon.

### Horizontal Gene Transfer: a source of gene innovation in metal transportomes

In view of the mosaic nature of genomes of the *Pseudomonas *spp we identified metal transporters with GI associated features from our inventory (see Additional files 6, 7, 8, 9). In our analysis we identified three distinct categories of transporters: those with anomalous composition (%G+C) alone, anomalous composition plus other GI associated features, normal %G+C plus GI associated features (Additional files 6, 7, 8 and 9). We identified thirty-two transporters (45.71%) in *Ppu *possessing highly significant GI features while they were twenty-six (48.10%) in *Psy*, nineteen (26%) in *Pfl *and seventeen (26%) in *Pae*.

Families with strong GI features that were present uniquely in a species were: CPA3 family (sodium ion homeostasis), ABC family (Cu transporter), P-type ATPase (Cu transporter) in *Pae*, NhaB family, NiCoT family in *Ppu*, NSS, MScS and CitMHS families in *Psy*. ABC transporter for Molybdenum was found to have GI features in the three *Pseudomonas *except for *Pfl*. Strong GI features were observed only for certain paralogs of metal transporter families indicating that paralog expansion could have occurred through HGT (Additional 10). These results show how different metal transporter families vary in their propensity for HGT among the four spp.

## Conclusion

In this article we performed a dataset cataloging for 262 metal transporters from four representative *Pseudomonas *species. This is the first comprehensive genomic comparison of metal transporters, providing potentially important insights into the fundamental molecular aspects and novel facets of *Pseudomonas *metal transportomes.

Our comparative inventory identified and analyzed novel metal transporters belonging to the following families: NiCoT (PP_2968, PSPTO_4280), Ca P-type ATPase (PA1429), Cu P-type ATPases (PP_4261, PA1549), ZIP (PP_1836, PP_0947, PFL_4718, PFL_0910, and PSPTO_2053) and MgtC (PP_3244, PP_3541, PA4635, PFL_4077, PFL_2871).

*Psy *possessing least number of metal transporters showed maximum percentage (48%) of transporters with strong GI features. Our data is substantiated by the previous observations where majority of *Psy *ORF's shared features with horizontally transferred genes [26]. *Ppu *has 45% of its transporters possessing strong GI features. On the contrary, *Pfl *and *Pae *have only 26% of their metal transporters exhibiting GI features.

This comparative inventory can therefore provide a window for *Pseudomonas *community in mining large and heterogeneous data sets obtained from metagenome projects to identify new biologically relevant patterns of metal transporters resembling those in this study. A combinatorial approach of transcriptomics and functional genomics will aid in deducing the functions of these diverse metal transporters and assembling a complete picture of metal homeostasis.

## Abbreviations

*Ppu*/PP/_*Ppu*_: *P.putida KT2440*; *Pae/*PA/_*Pae*_: *P.aeruginosa PA01*; *Pfl/*PFL/_*Pfl*_: *P.fluorescens Pf-5*; *Psy*/PSPTO/_*Psy*_: *P.syringae DC3000*; TMD's: Transmembrane domains; aa: Amino acid residues; bp: Base pairs; T.C.No: Transporter classification number; Me^2+ ^transporter: Hypothetical metal transporter; MFS: Major facilitator super family; IMP's: Integral membrane proteins; HGT: Horizontal gene transfer.

## Competing interests

The authors declare that they have no competing interests.

## Authors' contributions

AH performed the complete *insilico *analysis and wrote the draft of the manuscript.  AR contributed to critical evaluation of the work and improvement of the manuscript organization.  PMM conceived the study, guided the complete work and corrected the manuscript.

## Supplementary Material

Additional file 1 **Catalogue of *P.putida* KT2440 metal transporters.   **Description: This catalogue was compiled for the entire complement of alkali/alkaline earth metal transporters (Na+, K+, Ca2+, Mg2+), transition metal transporters (Zn2+, Mn2+ Cu2+, Ag+, Mo2+, Fe2+, Fe3+, Ni2+, Co2+) and heavy metal transporters (Cr3+, As3+, Pb2+, Cd2+) from *P.putida* KT2440. Information provided in this catalogue includes the class/family/subfamily, Transporter classification number (T.C.No),  TIGR  locus,  protein  name,  genomic  location, orientation,  length  of  the  protein, Transmembrane  domains  (TMD’s), substrate and the predicted role of all the metal transporters. Click here for file

Additional file 2 **Catalogue of *P.aeruginosa* PA01 metal transporters.**   Description: This catalogue was compiled for the entire complement of alkali/alkaline earth metal transporters (Na+, K+, Ca2+, Mg2+), transition metal transporters (Zn2+, Mn2+ Cu2+, Ag+, Mo2+, Fe2+, Fe3+, Ni2+, Co2+) and heavy metal transporters (Cr3+, As3+, Pb2+, Cd2+) from *P.aeruginosa* PA01. Information provided in this catalogue includes the class/family/subfamily, Transporter classification number (T.C.No),  TIGR  locus,  protein  name,  genomic  location, orientation,  length  of  the  protein, Transmembrane  domains  (TMD’s), substrate and the predicted role of all the metal transporters. Click here for file

Additional file 3 **Catalogue of *P.fluorescens* Pf-5 metal transporters.   **Description: This catalogue was compiled for the entire complement of alkali/alkaline earth metal transporters (Na+, K+, Ca2+, Mg2+), transition metal transporters (Zn2+, Mn2+ Cu2+, Ag+, Mo2+, Fe2+, Fe3+, Ni2+, Co2+) and heavy metal transporters (Cr3+, As3+, Pb2+, Cd2+) from *P.fluorescens* Pf-5. Information provided in this catalogue includes the class/family/subfamily, Transporter classification number (T.C.No),  TIGR  locus,  protein  name,  genomic  location, orientation,  length  of  the  protein, Transmembrane  domains  (TMD’s), substrate and the predicted role of all the metal transporters. Click here for file

Additional file 4 **Catalogue of *P.syringae* DC3000 metal transporters.**   Description: This catalogue was compiled for the entire complement of alkali/alkaline earth metal transporters (Na+, K+, Ca2+, Mg2+), transition metal transporters (Zn2+, Mn2+ Cu2+, Ag+, Mo2+, Fe2+, Fe3+, Ni2+, Co2+) and heavy metal transporters (Cr3+, As3+, Pb2+, Cd2+) from *P.syringae* DC3000. Information provided in this catalogue includes the class/family/subfamily, Transporter classification number (T.C.No),  TIGR  locus,  protein  name,  genomic  location, orientation,  length  of  the  protein, Transmembrane  domains  (TMD’s), substrate and the predicted role of all the metal transporters. Click here for file

Additional file 5 **Sequence Cluster Analysis for detection of paralogs from four *Pseudomonas spp*.**   Description:  This data provides evidence for the paralogous nature of metal transporters from various metal transporter families. Click here for file

Additional file 6 **Metal transporters exhibiting GI associated features from *P.putida* KT2440. **  Description: This table lists out the metal transporters with GI associated features from *P.putida* KT2440 and provides evidence for the metal transporters with strong and weak GI features. Click here for file

Additional file 7 **Metal transporters exhibiting GI associated features from *P.aeruginosa* PA01.**    Description: This table lists out the metal transporters with GI associated features from *P.aeruginosa* PA01 and provides evidence for the metal transporters with strong and weak GI features Click here for file

Additional file 8 **Metal transporters exhibiting GI associated features from *P.fluorescens*   Pf-5.**   Description: This table lists out the metal transporters with GI associated features from *P.fluorescens* Pf-5 and provides evidence for the metal transporters with strong and weak GI features. Click here for file

Additional file 9 **Metal transporters exhibiting GI associated features from* P.syringae *DC3000**.  Description: This table lists out the metal transporters with GI associated features from *P.syringae* DC3000 and provides evidence for the metal transporters with strong and weak GI features. Click here for file

Additional file 10 **Role  of  HGT  in  paralogous  expansion  and  acquisition  of  novel  transporter families in *Pseudomonas*. **  Description:  This  table  demarcates  the  role  of  HGT  in  paralogous  expansion  and acquisition of novel transporter families in each of the four *Pseudomonas* species. Click here for file
